# Characterization of Bioactive Phenolic Compounds Extracted from Hydro-Distillation By-Products of Spanish *Lamiaceae* Plants

**DOI:** 10.3390/molecules29225285

**Published:** 2024-11-08

**Authors:** Silvia Pérez-Magariño, Marta Bueno-Herrera, M. Carmen Asensio-S.-Manzanera

**Affiliations:** Agrarian Technological Institute of Castilla and León, Consejería de Agricultura y Ganadería, Ctra Burgos Km 119, Finca Zamadueñas, 47071 Valladolid, Spain; bueherma@itacyl.es (M.B.-H.); asesanmr@itacyl.es (M.C.A.-S.-M.)

**Keywords:** phenolic acids, flavonoids, terpenes, aromatic plants, solid residue

## Abstract

Plants of the *Lamiaceae* family are widely used for the extraction of essential oils, and this industry generates a large number of solid residues as by-products, which contain non-volatile valuable compounds. The aim of this work was to identify and quantify the phenolic compounds present in these solid residues from different important Spanish species of *Lamiaceae* to characterize and valorize them. Forty-seven phenolic compounds were identified by HPLC-DAD-MS and quantified by HPLC-DAD. Different concentrations and types of phenolic compounds were found between the solid residues. The *Rosmarinus officinalis* extracts showed the highest total phenolic content due to their high phenolic terpene concentrations. The *Thymus mastichina* extracts were characterized by kaempferol and flavanones, and some flavones were derived from luteolin and apigenin. Finally, the sample *Lavandula* and *Salvia lavandulifolia* extracts presented the lowest content of most phenolic compounds, with the exception of some phenolic acids, such as danshensu, salvianolic acid A, and glucosides of hydroxycinnamic acids. Therefore, this work provides information on the quantification of a large number of phenolic compounds using a simple, sensitive, reproducible, and accurate methodology. In addition, the results indicate that these solid residues still contain important amounts of different polyphenols, which are antioxidants and can be used in different industries.

## 1. Introduction

*Lamiaceae* is a cosmopolitan family of great diversity, comprising a large number of species. Species belonging to this family are commonly used as medicinal and/or aromatic plants (MAPs) because of their ability to synthesize volatile compounds and also because they are an important source of polyphenols. Plants of the *Lamiaceae* family are mainly used as ornamentals for the extraction of essential oils (EOs), for the food industry as nutraceuticals, and for phytotherapy and have increasing value and potential worldwide [1,2,3]. The global market for EOs was valued at USD 7.6 billion in 2018 and is expected to reach USD 15.1 billion by the year 2026, as evidence of the growing use of these products emerges worldwide [4].

EOs are usually extracted from raw plant material (flowers, buds, seeds, leaves, twigs, bark, herbs, wood, fruits and roots) by hydro-distillation or by steam distillation.

The EO yield is 0.5–8 *w*/*w* % of the dry plant, so the industrial processing of MAPs generates a large number of solid residues as by-products. Between 2017 and 2021, this industry generated more than 250 thousand tons of distillation residues per year [5]. Their disposal can lead to serious environmental problems, especially if they are incinerated or landfilled. Nowadays, the main challenge for the MAP sector is to add value to the by-products of the distillation and drying process [2]. Some industries attempt to valorize such waste by using them for energy or composting [6]. These recycling systems have some disadvantages. The industry requires a huge investment to recycle the by-products into energy. On the other hand, recycling for composting is not always satisfactory due to the antigermination properties of some MAPs, which can also be transferred from the plant residues. There is an increasing interest in recovering valuable bioactive compounds from the residues of the EO industry for their valorization [7]. These solid distillation residues still contain non-volatile valuable compounds, such as different polyphenols, which can be extracted and used as antioxidants in the food, feed, or cosmetic industries [8,9].

It is known that the distillation process can significantly affect the presence and number of different phenols in the solid residues, but nevertheless, they are an important source of phenolic compounds [6,10,11,12,13,14,15]. These compounds can be extracted using several solvents and their mixtures, such as methanol, ethanol, acetone, ethyl acetate, or water [14]. The nature and content of phenolic compounds varies widely between aromatic plants and species. The most important compounds in the *Lamiaceae* family are phenolic acids, flavonoids, and phenolic terpenes [16,17,18]. Therefore, the use of organic solvents should be necessary to favor the extraction of more non-polar compounds, mainly flavonoids and phenolic terpenes. Liquid Chromatography with Diode Array Detection and/or Mass Spectrometry is the most commonly used technique to evaluate and characterize the phenolic compounds of different plant materials [17,18,19]. The identification and quantification of phenolic compounds in plant material is a difficult task due to the complexity of polyphenolic structures, the large number of compounds that can be found, and the limited number of commercially available standards.

Therefore, most of the studies focus on the tentative identification of a large number of compounds using complex techniques with MS/MS or Q-TOF-MS detectors, but only some of them are focused on the quantification of these compounds. Moreover, these techniques require a high level of investment, infrastructure, and highly skilled personnel, which are beyond the reach of most industries and laboratories. The aim of this work was to identify and quantify as many as possible of the phenolic compounds present in the solid residue obtained after the EO extraction from different important Spanish species of *Lamiaceae* in order to characterize and add value to these residues. Six different aromatic plants were studied: *Thymus mastichina* L. (TM), *Salvia lavandulifolia* Vahl (SL), *Lavandula angustifolia* Mill. (LA), *Lavandula latifolia* Medik (LL), three types of *Lavandula x intermedia* Emeric ex Loisel. (LI_ Abrial, LI_Grosso and LI_Super), and *Rosmarinus officinalis* L. (RO). In addition, a simple analytical method using High-Performance Liquid Chromatography with Diode Array Detection (HPLC-DAD) and Mass Spectrometry (MS) was developed and validated. This work provides a simple methodology that allows, for the first time, the complete characterization and simultaneous quantification of 47 phenolic compounds in the by-products obtained from the distillation process of different Spanish MAPs. It has also highlighted the important amounts of bioactive phenolic compounds that can be extracted from these MAPs for use in different industries.

## 2. Results and Discussion

### 2.1. Identification of Phenolic Compounds in the Solid Residue from Hydro-Distillation of the Aromatic Plants

**Figure 1:**
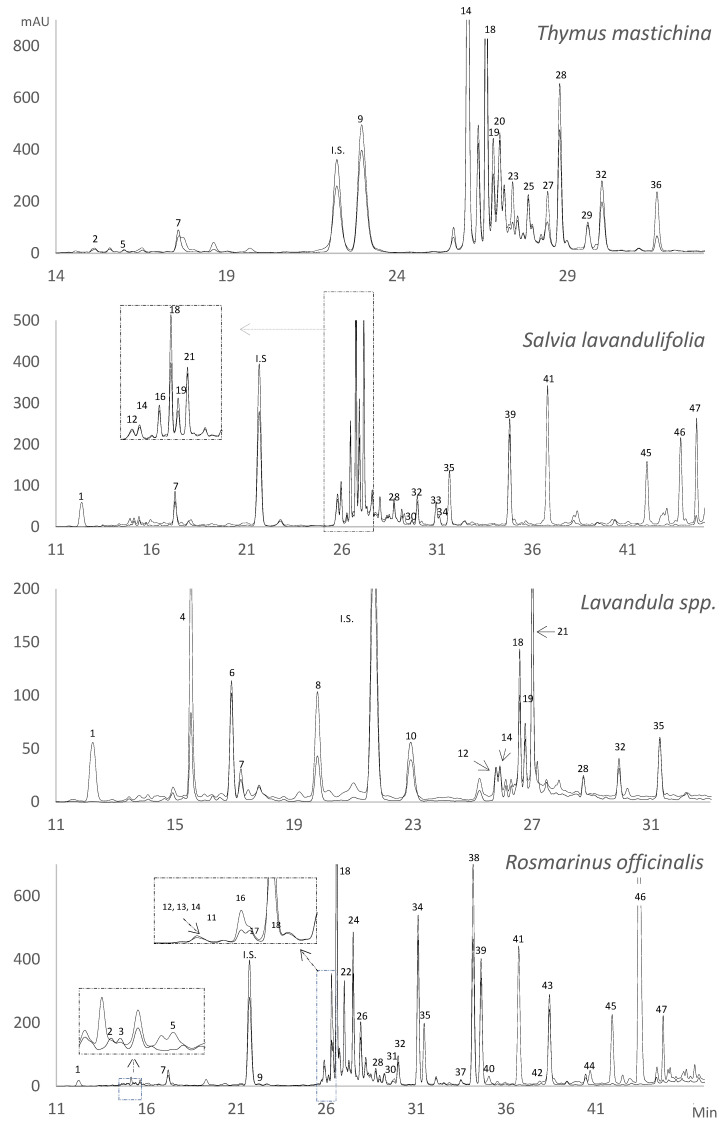
Chromatograms of the phenolic compounds of *Thymus mastichina*, *Salvia lavandulifolia*, *Lavandula* spp., and *Rosmarinus officinalis* monitored at 280 (dotted line) and 320 nm (continuous line). Peak numbers are the compounds shown in Table 1. IS: internal standard.

The extraction technique and the chromatographic method used allowed the characterization of forty-seven phenolic compounds, grouped into phenolic acids, flavonoids (flavones, flavanones, and flavonols), and phenolic terpenes. Table 1 summarizes the spectroscopic and the spectrometric data of all the identified phenolic compounds, numbered according to their elution order. Figure 1 shows the chromatograms of the different phenolic profiles of the *Lamiaceae* plants studied at two different wavelengths.

Twenty-nine compounds were identified by the standard compounds, and eighteen were tentatively identified from their UV absorption spectra and mass spectral data reported in the bibliography, as discussed below.

#### 2.1.1. Phenolic Acids

Twelve phenolic acids were identified, six by standard compounds (danshensu (3,4-dihydroxyphenyl lactic acid), chlorogenic acid, p-hydroxybenzoic acid, caffeic acid, rosmarinic acid, and salvianolic acid A) and another six were tentatively identified. Peak 3 had the same UV absorption spectrum as chlorogenic acid (UVmax 326 nm), with the same *m*/*z* 353 and fragments. It was identified as crytochlorogenic acid, which was also supported by Irakli et al. [18], who identified this compound by its standard and it was detected in RO.

Peak 4 presented the molecular ion *m*/*z* 325 and a fragment ion at 163 *m*/*z* corresponding to the coumaric acid and a loss of 162 amu that reveals the presence of glucose or galactose. However, no galactoside compounds of hydroxycinnamic acids have been found in the literature, so this glycoside is most probably glucoside [20], and therefore, this compound was tentatively identified as coumaric acid-O-glucoside [8,17]. Similarly, peaks 6 and 10 were identified as glucosides of ferulic acid since they presented the molecular ion at *m*/*z* 355 and fragment ions at *m*/*z* 193, corresponding to the ferulic acid with a loss of glucose. These two compounds were also identified by Torras-Claveria et al. [8] and Contreras et al. [17] in the genus *Lavandula* and other *Lamiaceae* species, respectively. Other glucosides of hydroxycinnamic acids identified were dihydro-p-coumaric acid glucoside or hydroxyhydrocinnamic acid glucoside (peak 8) found in the genus *Lavandula* and rosmarinic acid-3-O-glucoside (peak 11) in RO. The mass fragmentation data of peak 8 showed the molecular ion at *m*/*z* 327 and a fragment ion at *m*/*z* 165, indicating the loss of glucose. 

These data agree with those found by Contreras et al. [17], who identified this compound in *Helleborus niger*. Rosmarinic acid-3-O-glucoside presented the molecular ion *m*/*z* 521, and it was also identified by Borras-Linares et al. [21] and Sharma et al. [22] in RO.

#### 2.1.2. Flavones and Flavonols

Twenty-one flavones were identified, of which fourteen were standard compounds (luteolin-7-O-rutinoside, luteolin-7-O-glucuronide, luteolin-7-O-glucoside, nepetin-7-O-glucoside (nepitrin), apigenin-7-O-glucoside, scutellarein, luteolin, cirsiliol, apigenin, eupatorine, cirsimaritin, acacetin, genkwanin and salvigenin) and seven were tentatively identified. All of the flavones were derivatives of apigenin and luteolin. Peaks 9, 20, and 26 were luteolin derivatives. Peak 9 was identified as 6-hydroxyluteolin-7-O-glucoside and presented molecular ions at *m*/*z* 463 and a fragment ion at *m*/*z* 301 due to the loss of a glucose moiety. This compound was found in TM and RO and was also identified by Borras-Linares et al. [21] and Sharma et al. [22] in RO. Peak 20 presented the ion *m*/*z* 301 and the same UV absorption spectrum as luteolin, and it was tentatively identified as 6-hydroxyluteolin, being identified only in TM. Peak 26 had a molecular ion at *m*/*z* 503 and fragment ions at *m*/*z* 399 and 285. This fragmentation pattern agreed with one of the isomers of luteolin-3’-O-(O-acetyl)-β-D-glucuronide identified in RO by Borras-Linares et al. [21], Mena et al. [16], Achour et al. [23], and Sharma et al. [22] and it could be luteolin-3’-O-(2″-O-acetyl)-β-D-glucuronide [21,23]. 

Peaks 22, 24, 35, and 43 were apigenin derivatives, all of which were identified in RO, and peak 35 was also identified in SL and the genus *Lavandula*. Peaks 22 and 24 presented the molecular ions at *m*/*z* 461 and different fragment ions at *m*/*z* 299 and 285, respectively.

The first peak (peak 22) was identified as hispidulin-7-glucoside, with the fragment ion *m*/*z* 299 corresponding to hispidulin with a loss of glucose (162 amu), also called homoplantaginin according to the data found in the bibliography [18,22,24]. 

Peak 24 was identified as scutellarin or scutellarein-7-glucuronide, with the fragment ion *m*/*z* 285 corresponding to scutellarein with a loss of glucuronide acid (176 amu), as was previously reported by Sharma et al. [22].

The molecular ion of peak 35 was only found in the positive mode with *m*/*z* 315, which may correspond to ladanein, a dimethyl scutellarein derivative, with methylations at 4′ and 7 hydroxyls [25,26]. According to Psarrou et al. [26], this compound showed two maximum absorbances at 286 and 334 nm, which agrees with the data in our study (Table 1). This pattern of the protonated molecular ion [M-H] + was also found in two other flavones, eupatorin and salvigenin as compounds for which a standard was available. Peak 43 also presented an apigenin-type UV spectrum with maximum absorbances at 268 and 332 nm with a molecular ion at *m*/*z* 299 in the positive mode. This compound was identified as 4′-methoxytectochrysin or apigenin-4′,7-dimethylether, taking into account the data found in the bibliography and the fact that it was the only flavonoid that elutes between carnosol and carnosic acid [19,25,26,27].

Five flavanones were identified by their respective standards, naringenin, naringenin-7-O-glucoside, eriodictyol, sakuranetin, and hesperidin. The first four compounds were only detected in the TM extracts, and hesperidin was only detected in the SL and RO extracts.

Kaempferol was identified by its standard, and it was the only flavonol detected in the TM extracts.

#### 2.1.3. Phenolic Terpenes

Finally, the phenolic terpenes were detected in the solid residue of RO and SL. Carnosic acid is known to be unstable, and its degradation is favored by temperature, exposure to light, and the presence of oxygen. Under these conditions, carnosic acid can be oxidized to carnosol, rosmanol, epirosmanol, epiisorosmanol, methylepirosmanol, rosmadial, and methylcarnosate, among others [28,29,30]. Bicchi et al. [31] and Troncoso et al. [32] reported that carnosol is the main compound obtained after the degradation of carnosic acid, which can be transformed into other phenolic diterpenes such as rosmanol and its isomers, epirosmanol, and epiisorosmanol. In addition, the use of methanol in the extraction and under acid conditions could break the lactone bond of carnosol, giving rise to other compounds derived from carnosic acid, and carnosol [29].

In this study, eight phenolic terpenes were identified: three by standards (rosmanol, carnosol and carnosic acid), and the other five were tentatively identified. All these compounds showed similar UV spectra to carnosic acid and carnosol with maximum absorbances at 284 nm, so they are derived from carnosic acid.

Peak 40 presented the molecular ion at *m*/*z* 359 and a fragment ion at *m*/*z* 283, corresponding to the loss of a methoxy (-O-CH_3_) and an acid (-COOH) group. This compound was identified as rosmanol methyl ether or epirosmanol methyl ether according to Borras-Linares et al. [21], Mena et al. [16], and Sharma et al. [22]. This compound can be formed from carnosol due to the use of methanol for extraction, which agrees with Doolaege et al. [29]. Zhang et al. [30] obtained the corresponding ethyl ether of epirosmanol as a degradation product of carnosol when carnosol was dissolved in ethanol. These degradation products were not formed when an aprotic solvent such as acetonitrile was used.

Peak 42 had a molecular ion at *m*/*z* 343 and a fragment ion at *m*/*z* 299 due to the loss of an acid group (-COOH), and a maximum absorbance at 288 nm. These data agreed with those found in the bibliography [21,22,30] and it was identified as rosmadial.

Peaks 44 and 47 also presented UV absorption spectra similar to carnosol and carnosic acid and molecular ions at *m*/*z* 329 and 345, respectively, which were identified as carnosol isomer and 12-methylcarnosic acid, according to other studies [21,22]. The carnosol isomer showed the same fragmentation pattern as carnosol (with molecular ions at *m*/*z* 329 and a fragment ion at *m*/*z* 285). On the other hand, 12-methylcarnosic acid had two fragment ions (*m*/*z* 301 and 286) corresponding to the loss of the acid group (44 amu) and the subsequent loss of the methyl group (15 amu).

Finally, peak 45 presented a molecular ion at *m*/*z* 301 and showed a UV absorption maximum at 280 nm. Different authors have reported a compound with these mass and UV spectral characteristics, but they have assigned different structures. Borras-Linares et al. [33] identified it as salviol, which is an abietatriene compound with a single phenol group isolated from some Salvia species. Doolaege et al. [29] identified and characterized one compound with a molecular weight of 302, which is a fragment ion of *m*/*z* 301 and has a UV spectrum similar to carnosic acid. They hypothesized that this compound results from the breakdown of the intramolecular bond of rosmanol and its isomers under acid conditions, followed by the formation of an unsaturated double bound and the loss of a CO-group. The UV spectrum of this compound had an absorbance pattern similar to that of our peak 45. Finally, Zhang et al. [30] proposed a structure that was named 5,6,7,10-tetrahydro-7-hydroxy-rosmariquinone, which was a major degradation product derived from carnosic acid. In all these studies, as in our work, this compound eluted before carnosic acid. With the data available in this study, we could not confirm the structure of this compound, but it was clear that it was derived from carnosic acid. Therefore, this compound has been named a carnosic acid derivative.

Finally, two rosmanol isomers, epiisorosmanol, and epirosmanol, were identified by their MS fragmentation patterns. They presented the same molecular ion at *m*/*z* 345 as rosmanol, but they had some differences in the fragmentation pattern and eluted at different retention times. According to Kontogianni et al. [24], Achour et al. [23], and Sharma et al. [22], the first isomer, epiisorosmanol, eluted before rosmanol and showed the major fragment ions at *m*/*z* 301 and 283, corresponding to the loss of 44 amu (acid group) and later of 18 amu (water molecule). The second, epirosmanol, eluted after rosmanol and showed only the major fragment at *m*/*z* 283. These isomers were clearly identified by MS. However, they were not quantified due to the low signal found in the DAD.

### 2.2. Results of the Method Validation

Table 2 shows the range, calibration curve, correlation coefficients, LOD, and LOQ for each standard phenolic compound. The linearity in the range indicated that all compounds were satisfactory, with correlation coefficients between 0.9952 and 0.9999. The LOD and LOQ values indicated that the developed method had sufficient sensitivity for the analysis of phenolic compounds in these aromatic plant residues.

The repeatability and reproducibility values were satisfactory, ranging from 0.8 to 7.3% for repeatability and from 2.3 to 9.4% for reproducibility, being less than 10% for all the phenolic compounds (Table 3).

In general, good recoveries were obtained, ranging from 80 to 120% for most of the compounds. Only kaempferol, carnosol, and carnosic acid showed recoveries outside this range. Kaempferol showed recoveries of around 65%, which may be due to the fact that this compound is very reactive and may react with other phenolic compounds [34]. Similarly, carnosol and carnosic acid showed recoveries of around 135% and 67%, respectively. This may be due to the fact that carnosic acid is unstable and can be oxidized to carnosol and other related compounds [28,29,30], as previously commented. Furthermore, carnosol is the main compound obtained after carnosic acid degradation [31,32], which is consistent with the loss of about 33% of carnosic acid and the increase to 33% of carnosol.

Therefore, all these results indicate that this method can be considered precise, reproducible, and accurate.

### 2.3. Quantification of Phenolic Compounds in the Solid Residue from Hydro-Distillation of the Aromatic Plants

Table 4 shows the concentration of the phenolic compounds quantified in the solid residue of the EO by-product of the different *Lamiaceae* aromatic plants studied. The methodology described above allowed the identification and quantification of forty-seven phenolic compounds. However, not all the compounds were found in the solid residue of the different plants. Only five compounds, caffeic acid, rosmarinic acid, luteolin-7-O-glucoside, luteolin, and apigenin, were found in all the studied by-products.

High differences were found between the solid residues from the *Lamiaceae* aromatic plants evaluated. The RO extracts showed the highest total phenolic content (56,459 ± 1375 µg/g) due to the high phenolic terpene concentrations. The extracts from TM and SL showed a medium and similar concentration of phenolics (10,002 ± 3193 µg/g and 9301 ± 11,089 µg/g, respectively), although the type of phenolics present in each plant was different. The TM extracts did not contain any phenolic terpenes and were characterized by a higher content of flavones and flavanones in comparison to the SL extracts. The SL extracts were mainly characterized by the presence of phenolic terpenes (46%), followed by phenolic acids (30%) and flavones (24%). Finally, the extracts from *Lavandula* spp. had the lowest total phenolic content of all the extracts studied (average of 2825 µg/g) and contained only phenolic acids and flavones.

**Figure 2:**
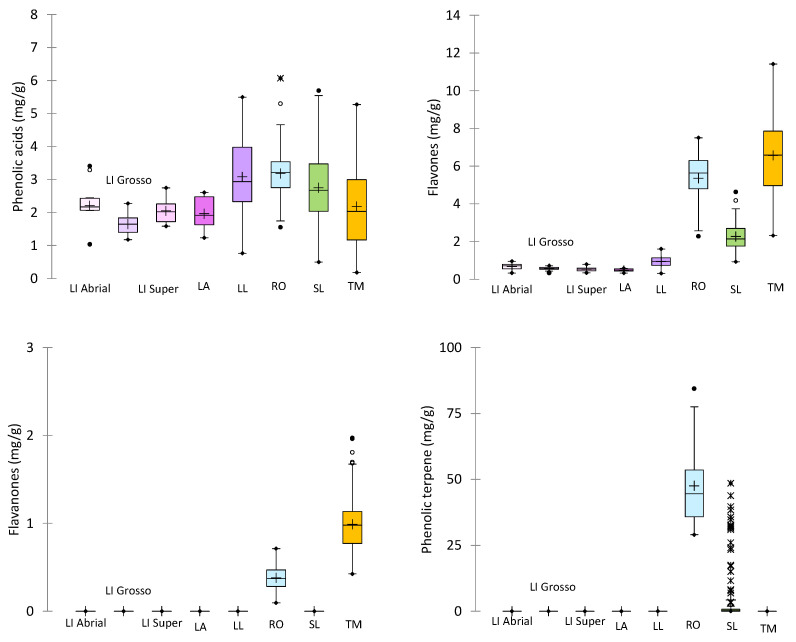
Box and Whisker plot of the different phenolic families of the solid residues from different Spanish species of *Lamiaceae*. +: mean values; ●, ○ and 🞻: outliers.

It is noteworthy that differences in the content of phenolic compounds were found between the samples (Figure 2). Although all samples were collected in the same locality, they belonged to several seasons, and different weather conditions can affect the chemical composition. Furthermore, the variability was higher in wild species (TM, SL, LL, and RO) than in those that come from selected clones (*Lavandula* spp.) (Table 4, Figure 2). Variations in LA and lavandin were exclusively due to the weather conditions between years, while different populations from different locations were used to set the cultivation trials in wild species.

Rosmarinic acid was the major phenolic acid present in the aromatic plants studied as an ester obtained from the esterification product of 3,4-dihydroxyphenyl lactic acid (danshensu) and caffeic acid [35]. The extracts from RO and TM were the richest in rosmarinic acid, followed by the SL extracts. On the other hand, the solid residue of *Lavandula* spp presented the lowest concentration of rosmarinic acid (between 3 and 10 times lower than the content of the other plant extracts studied) but the highest concentration of danshensu, which is the precursor of rosmarinic acid. Salvianolic acid A was found in SL and *Lavandula* spp. extracts, which are formed by one molecule of danshensu and two molecules of caffeic acid [36]. Different salvianolic acids were found in high concentrations in *Salvia miltiorrhiza* (Danshen), which is a plant extensively used in traditional Chinese medicine for the treatment of cardiovascular diseases due to its antioxidant and free radical scavenging activities [37,38]. The rosmarinic acid glucoside was only found in the RO extract and is characteristic of this plant [21]. The hydroxycinnamic acid derivatives of coumaric and ferulic acids were only detected in *Lavandula* spp. extracts.

The flavonoids were the predominant compounds quantified in TM extracts at 78%. Among the flavonoids, the flavones were the most abundant phenolic group (66%), mainly formed by the aglicones luteolin and apigenin, 6-hydroxyluteolin, 6-hydroxyapigenin, and their O-glycoside derivatives. On the other hand, the RO extracts were characterized by the methyl-ether, methoxy, and glucuronide derivatives of luteolin and apigenin. Among the flavones, the SL extracts stood out for their content of salvigenin, apigenin-7-O-glucoside, and cirsimaritin, and the *Lavandula* spp. extracts for their content of luteolin-7-O-glucoside, apigenin-7-O-glucoside, and luteolin-7-O-rutinoside. In addition, as previously mentioned, kaempferol was the only flavonol found in TM, and it was not detected in the other solid residues of the MAPs analyzed.

The RO extracts were highlighted for their high phenolic terpene content, accounting for 84% of the total amount of phenolic compounds, with carnosic acid being the most abundant, followed by carnosol, carnosic acid derivative, and 12-methylcarnosic acid. Carnosic acid is a labdane-type diterpene with a strong antioxidant activity, but it is also very unstable in the presence of oxygen [33]. As a result, other phenolic terpenes are formed from the degradation of carnosic acid, including carnosol, methyl carnosate, rosmanol, and rosmadial [29]. Kontogianni et al. [24] attributed the antioxidant activity of fresh RO extracts to the presence of carnosol, rosmarinic acid, and carnosic acid. In addition to the RO extracts, only the SL extracts showed a phenolic terpene content of 46% of the total phenols, but quantitatively, it was 10 times lower than the RO extracts.

A principal component analysis (PCA) was performed to investigate whether the information given by the composition in the phenolic compounds could allow the solid residues obtained from the distillation of the MAPs to be differentiated. The PCA selected four components that explained 75.1% of the total variance (Table S1). 

**Figure 3:**
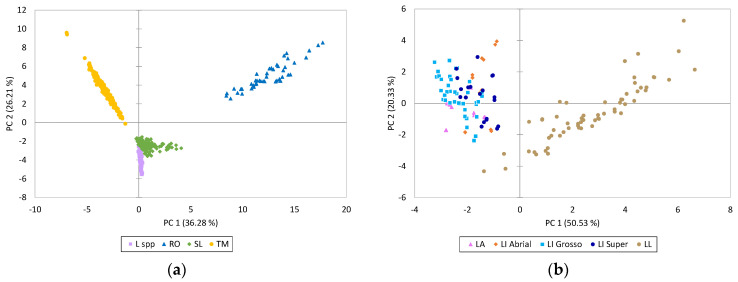
Principal component analysis. (**a**) Representation of the samples of solid residues of *Thymus mastichina* L. (TM), *Salvia lavandulifolia* Vahl (SL), *Lavandula* spp. (L. spp.), and *Rosmarinus officinalis* L. (RO); (**b**) representation of the samples of solid residues of *Lavandula latifolia* Medik (LL), three types of *L. x intermedia* Emeric ex Loisel. (LI_ Abrial, LI_Grosso and LI_Super), and *L. angustifolia* Mill. (LA).

Figure 3a shows the distribution of the samples in the plane defined by the first two dimensions that explained 62.5% of the total variance. As can be observed, all the differences described above in the phenolic profile allowed us to distinguish the solid residues obtained from the distillation of the studied plant species. The RO extracts, cited for the positive side of PC 1 and PC 2, were clearly separated from the other extracts and were positively correlated with the phenolic terpenes; some flavones (mainly glucuronide derivatives of luteolin and apigenin, hispidulin-7-O-glucoside and nepetin-7-O-glucoside); and cryptochlorogenic acid and rosmarinic acid-3-O-glucoside (Table S1). On the other hand, the TM extracts were located on the upper left side of the plane, correlating mainly with kaempferol and flavanones, and some flavones such as aglicones luteolin and apigenin, 6-hydroxyluteolin, 6-hydroxyapigenin, and their O-glycoside derivatives. Finally, the samples belonging to species of the genus *Lavandula* were grouped together and closer to the *S. lavandulifolia* samples, indicating that these two species are closer in terms of chemotaxonomy. These samples were located on the lower side of the plane due to the lowest content of most of the phenolic compounds, with the exception of the phenolic acids, danshensu, salvianolic acid A, coumaric acid-O-glucoside, dihydro-p-coumaric acid glucoside, and two ferulic acid-O-glucosides, and the flavone, luteolin-7-O-rutinoside (Table S1).

Another PCA was performed only with the *Lavandula* spp. samples, and it selected three components that explained 78.3% of the total variance (Table S2). Figure 3b shows the distribution of the *Lavandula* spp. samples in the plane defined by the first two dimensions that explain 70.9% of the total variance. Among the *Lavandula* spp. samples, spike lavender (LL), sited on the right side of the plane, was separated from lavender and lavandin, which were grouped together. The LL extracts were positively correlated with the phenolic acids, danshensu, coumaric acid-O-glucoside, dihydro-p-coumaric acid glucoside, rosmarinic acid, and salvianolic acid A, and the flavones, luteolin-7-O-glucoside, and apigenin-7-O-glucoside (Table S2). Although the concentrations of each phenolic compound can be affected by the population origin or the environmental conditions in which the plants grow, the phenolic profile of the species is preserved. Therefore, chemotaxonomy has been widely used in the *Lamiaceae* family in different genera and has even been used to differentiate taxa at the subspecies level [39,40].

## 3. Materials and Methods

### 3.1. Plant Samples

Six different aromatic plants were studied: *Thymus mastichina* L. (TM), *Salvia lavandulifolia* Vahl (SL), and *Lavandula latifolia* Medik (LL) and three types of *L. x intermedia* Emeric ex Loisel, including (LI_ Abrial, LI_Grosso and LI_Super), *L. angustifolia* Mill. (LA), and *Rosmarinus officinalis* L. (RO). All the samples analyzed came from cultivation trials located in the experimental farm of ITACyL in Valladolid. The plant material consisted of several wild populations of TM, SL, LL, and RO (8 to 15 different populations depending on the species) and was previously collected with vegetation multiplied around the Iberian Peninsula. LA, LI_ Abrial, LI_Grosso, and LI_Super plant materials were supplied by a commercial nursery. Samples were collected in different assays from 2013 to 2021. A total of 576 samples from different species and years were analyzed.

The aerial parts of wild and/or cultivated populations of *Lamiaceae* species were collected during the blossom phase in different years. The plant material was dried at room temperature in the dark for four weeks after collection. When the drying process was finished, leaves and flowers were separated from the stems, and only the mixture of leaves and flowers was used for distillation. The plant material after the distillation process was used for analysis.

### 3.2. Chemicals

HPLC-grade reagents and analytical-grade reagents were provided by Panreac (Madrid, Spain). The standard phenolic compounds were purchased from Sigma–Aldrich (Steinheim, Germany), Fluka (Buchs, Switzerland), Phytolab (Vestenbergsgreuth, Germany), Extrasynthèse (Lyon, France), and Targetmol (Wellesley Hills, MA, USA), as has been indicated in Table 2. P-coumaric acid was used as the internal standard (IS) and was provided by Sigma–Aldrich (Steinheim, Germany).

### 3.3. Solid Residue from the Hydro-Distillation of the Aromatic Plants and Extraction of the Phenolic Compounds

In total, 150 g of each dry sample was hydro-distilled (2 L) for 2.5 h using a Clevenger-type apparatus to extract the EOs. The solid residue obtained after the distillation was dried in an oven at 30 °C ± 2 °C for about 48 h. The dry solid residue was ground using a Retsch ZM 1 centrifugal mill (Fisher Scientific S.L., Madrid, Spain) and sieved between 800 and 280 µm.

In total, 0.25 g of the dry solid residue was extracted four times with 10 mL of methanol at room temperature by shaking in an orbital shaker KS-15 A (Bühler, Switzerland) at 150 rpm for 30 min. In the first extraction, 400 µL of p-coumaric acid was added as an internal standard (IS, 400 mg/L). The four extracts were pooled and filtered through nylon membrane filters at 0.45 μm, 47 mm (Filter-Lab, Barcelona, Spain). The extracts of each sample were concentrated under vacuum at 35 °C using a rotary evaporator R-210 (Büchi, Flawil, Switzerland) to a volume of less than 5 mL. This concentrated extract was transferred to a 5 mL volumetric flask and made up to the mark with methanol. The extracts were filtered (PVDF filters, 0.45 μm) and injected into the chromatograph on the day of extraction.

### 3.4. High-Performance Liquid Chromatography Diode Array Detection (HPLC-DAD) for the Quantification of Phenolic Compounds

The filtered extracts obtained were analyzed with an Agilent Technologies LC series 1200 (Boblingen, Germany) equipped with a quaternary gradient pump, a thermostated column compartment, an automatic injector, and a Diode Array Detection (DAD) system. The chromatographic separation was achieved on a reverse-phase ACE C18 column (250 mm × 4.6 mm i.d., 5 μm particle size) provided by Symta (Madrid, Spain) and thermostated at 25 °C. The mobile phase was solvent A (20 mM formic acid buffer pH = 2.8) and solvent B (methanol). The gradient was linear at a flow rate of 0.8 mL/min: 0 min, 10% B; 5 min, 20% B; 8 min, 38% B; 18 min, 38% B; 20 min, 70% B; 37 min, 90% B; and 38 min, 100% B and then held for 7 min before returning to the initial conditions.

The injection volume of samples and standards was 100 μL, using an injection program mixing 80 μL of solvent A and the 20 μL sample. The UV spectra were recorded in the range of 200–400 nm range, and chromatograms were recorded at 254, 280, 320, and 350 nm.

The quantification was carried out at different wavelengths depending on each compound (Table 1), using the internal standard method. The compounds tentatively identified where commercial standard was available and were quantified using the calibration curve of the available standard with the most similar UV absorption spectrum belonging to the same phenolic class: chlorogenic acid for peak 3, trans-cinnamic acid for peaks 4 and 8, ferulic acid for peaks 6 and 10, rosmarinic acid for peak 11, luteolin for peaks 9, 20, and 26, apigenin for peaks 22, 24, 35, and 43, and rosmanol for peaks 40 and 42 and carnosic acid for peaks 44, 45, and 47. The phenolic compound contents were expressed in micrograms per gram of dry solid residue weight.

### 3.5. Liquid Chromatography Diode Array Detection and Mass Spectrometer (LC-DAD-MS) for the Identification of Phenolic Compounds

The identification of the phenolic compounds was carried out on an Agilent LC series 1200 (Boblingen, Germany) equipped with a binary pump, a thermostated column compartment, an automatic injector, a 1260 DAD system, and a Mass Spectrometer Series 6410 Triple Quad. The chromatographic conditions were the same as for HPLC-DAD analyses, except that the flow rate was reduced to 0.6 mL/min. An electrospray ionization (ESI) source was used, and the ESI conditions were as follows: capillary voltage, 4 kV; nebulizer pressure, 45 psi; drying gas flow, 8 L/min; and drying gas temperature, 350 °C. Full mass spectra were recorded in both positive and negative ionization modes in the range of m/z 100–1000, using a cone voltage of 150 V. 

The compounds without standards were identified tentatively by comparing the UV spectra characteristics and the mass spectra (molecular weight (*m*/*z*) and ion fragmentation) with data available in the literature. Most of the compounds were clearly recognized from the negative ESI-MS spectra, but in some cases, they were identified in the positive mode.

### 3.6. Method Validation

Calibration, precision, accuracy, limits of detection, and quantification were evaluated for method validation.

The internal standard calibration curves were made using the corresponding phenolic standards at twelve different concentrations, and the linearity and range were determined for each phenolic compound. 

The precision was calculated by performing three different extractions of each sample on the same day (repeatability, %RSD, n = 3) and on three different days (reproducibility, %RSD, n = 9), and injecting on each day of the extraction.

The accuracy was calculated by spiking two different samples with known amounts of some standards at two different concentrations and analyzing these samples twice a day on three different days for each added concentration to determine the recovery percentage (% RSD, n = 6). Therefore, the matrix effect was considered.

The limit of detection (LOD) is the lowest concentration at which the instrument can detect but not quantify, and the limit of quantification (LOQ) is the lowest concentration at which the instrument can detect and quantify each compound. These limits were calculated from the standard deviation of the y-interceptions of the regression lines (Sy) and the slope of the calibration curve (S) at levels approaching the LOD (LOD = 3.3 × Sy/S and LOQ = 10 × Sy/S).

### 3.7. Statistical Analyses

The concentrations of each phenolic compound were expressed as the mean ± standard deviation (SD) in the solid residues from each MAP. A Box and Whisker Plot were used to represent the data distribution between the solid residues from different Spanish species of *Lamiaceae*.

Principal component analysis (PCA) was carried out to determine similarities or differences between the concentration of the phenolic compounds evaluated in the solid residue of the MAPs studied. 

All the statistical analyses were performed using the XLSTAT software (Addinsoft, version 2022.1).

## 4. Conclusions

A simple, sensitive, reproducible, and accurate HPLC-DAD-MS method is proposed that allows the simultaneous identification and quantification of forty-seven phenolic compounds grouped into phenolic acids, flavonoids (flavones, flavanones, and flavonols), and phenolic terpenes in the solid residue obtained after the EO extraction from different important Spanish species of *Lamiaceae*. The *Rosmarinus officinalis* extracts showed the highest total phenolic content due to the high concentrations of phenolic terpenes. The *Thymus mastichina* extracts were characterized by kaempferol and flavanones, and some flavones derived from luteolin and apigenin. Finally, the samples belonging to species of the genus *Lavandula* were closer to the *Salvia lavandulifolia* extracts, presenting the lowest content of most of the phenolic compounds, with the exception of some phenolic acids, such as danshensu, salvianolic acid A, and glucosides of hydroxycinnamic acids, and the flavone, luteolin-7-O-rutinoside. These results indicate that these solid residues still contain important amounts of different polyphenols, which are antioxidants and can, therefore, be used in different industries.

Therefore, this work provides information on the quantification of a large number of phenolic compounds using a simple methodology and the characterization and valorization of the solid residues obtained from the distillation process of EOs.

## Figures and Tables

**Table 1:** Peak number, spectroscopic and spectrometric data, and quantification wavelength (QW) of the phenolic compounds identified in the solid residue of the EO by-product of *Lamiaceae* aromatic plants.

Peak	Compound	Phenolic Class	λ Max (nm)	[M-H]^−^	Main Fragments	QW (nm)	IdC ^b^	TM ^c^	SL ^c^	L ^c^	RO ^c^
1	Danshensu (3,4-dihydroxyphenyl lactic acid)	Phenolic acid	282	197	179, 135	280	S		X ^e^	X	X
2	Chlorogenic acid	Phenolic acid	300sh ^a^, 326	353	191, 179	320	S	X			X
3	Cryptochlorogenic acid	Phenolic acid	300sh, 326	353	191, 179	320	T				X
4	Coumaric acid-O-glucoside	Phenolic acid	264, 296sh	325	163, 119	254	T			X	
5	p-hydroxybenzoic acid	Phenolic acid	256	137		254	S	X			X
6	Ferulic acid-O-glucoside 1	Phenolic acid	282sh, 304	355	193, 149	320	T			X	
7	Caffeic acid	Phenolic acid	298sh, 320	179	135	320	S	X	X	X	X
8	Dihydro-p-coumaric acid glucoside	Phenolic acid	276, 314sh	327	165, 121	280	T			X	
9	6-hydroxyluteolin-7-O-glucoside	Flavone	282, 344	463	301	350	T	X			X
10	Ferulic acid-O-glucoside 2	Phenolic acid	294, 320	355	193, 149	320	T			X	
11	Rosmarinic acid-3-O-glucoside	Phenolic acid	288sh, 322	521	359	320	T				X
12	Luteolin-7-O-rutinoside	Flavone	254, 266, 350	593	285	350	S		X	X	X
13	Luteolin-7-O-glucuronide	Flavone	258, 266, 350	461	285	350	S				X
14	Luteolin-7-O-glucoside	Flavone	258, 268, 350	447	285	350	S	X	X	X	X
15	Naringenin-7-O-glucoside	Flavanone	284, 332sh	433	271	280	S	X			
16	Hesperetin-7-rutinoside (hesperidin)	Flavanone	284, 334sh	609	301	280	S		X		X
17	Nepetin-7-O-glucoside (nepitrin)	Flavone	258, 272, 348	477	315	350	S				X
18	Rosmarinic acid	Phenolic acid	290sh, 330	359	197, 179, 161	320	S	X	X	X	X
19	Apigenin-7-O-glucoside	Flavone	268, 336	431	269	320	S	X	X	X	
20	6-hydroxyluteolin	Flavone	252–268, 350	301		350	T	X			
21	Salvianolic acid A	Phenolic acid	288, 310sh, 336sh	493	295	280	S		X	X	
22	Hispidulin-7-O-glucoside	Flavone	276, 334	461	299	350	T				X
23	Eriodictyol	Flavanone	288, 334sh	287	151, 135	280	S	X			
24	Scutellarein-7-O-glucuronide	Flavone	270, 338	461	285	350	T				X
25	Scutellarein	Flavone	286, 338	285		350	S	X			
26	Luteolin-3′-acetyl-O-glucuronide	Flavone	270, 338	503	399, 285	350	T				X
27	Naringenin	Flavanone	290, 332sh	271		280	S	X			
28	Luteolin	Flavone	254, 266, 350	285		350	S	X	X	X	X
29	Kaempferol	Flavonol	266, 366	285		350	S	X			
30	Cirsiliol	Flavone	256–274, 346	329		350	S		X		X
31	Rosmanol	Phenolic terpene	290	345	283	280	S				X
32	Apigenin	Flavone	268, 340	269		350	S	X	X	X	X
33	Eupatorin	Flavone	276, 344	345 ^d^		350	S		X		
34	Cirsimaritin	Flavone	274, 336	313	298, 283	350	S		X		X
35	Ladanein	Flavone	284, 334	315 ^d^		350	T		X	X	X
36	Sakuranetin	Flavanone	288, 330sh	285		280	S	X			
37	Acacetin	Flavone	268, 334	283		350	S				X
38	Genkwanin	Flavone	268, 338	283		350	S				X
39	Salvigenin	Flavone	276, 332	329 ^d^	287 ^d^	320	S		X		X
40	(Epi)rosmanol methyl ether	Phenolic terpene	290	359	283	280	T				X
41	Carnosol	Phenolic terpene	284	329	285	280	S		X		X
42	Rosmadial	Phenolic terpene	284	343	299, 285	280	T				X
43	4′-methoxytectochrysin	Flavone	268, 332	299 ^d^		350	T				X
44	Carnosol isomer	Phenolic terpene	284	329	285	280	T				X
45	Carnosic acid derivative	Phenolic terpene	280	301		280	T				X
46	Carnosic acid	Phenolic terpene	286	331	287	280	S		X		X
47	12-methylcarnosic acid	Phenolic terpene	284	345	301, 286	280	T				X

^a^ sh: shoulder. ^b^ IdC: identification compound. S: standard compound; T: tentatively identified compound with data reported in the literature. ^c^ TM: *Thymus mastichina*; SL: *Salvia lavandulifolia*; L: *Lavandula* spp.; RO: *Rosmarinus officinalis*. ^d^ [M-H]^+^. ^e^ compounds that appear in the different MAPs.

**Table 2:** Range, linearity, correlation coefficient (R^2^), sensibility data, and accuracy of the standard phenolic compounds.

Compound	Range (µg/mL)	Calibration ^a^	R^2^	LOD ^b^ (µg/mL)	LOQ ^b^ (µg/mL)	Supplier ^c^	Amounts Added (µg/mL)	Recovery (% RSD, *n* = 6)	
								TM	SL
Danshensu	2.0–120	y = 0.0044x + 0.0012	0.9984	1.1	4.3	1	24/48	-	97/90
Chlorogenic acid	1.0–10	y = 0.0130x − 0.0006	0.9973	0.37	1.1	1	1/2	102/100	
*p*-hydroxybenzoic acid	0.94–28	y = 0.0427x + 0.0129	0.9996	0.07	0.92	2	1/2	94/94	
Caffeic acid	1.0–31	y = 0.0223x − 0.0010	0.9987	0.70	2.2	1	4/8	105/106	120/112
Luteolin-7-O-rutinoside	1.3–60	y = 0.0093x − 0.0009	0.9997	0.78	2.3	1	4/8		102/95
Luteolin-7-O-glucuronide	2.0–160	y = 0.0122x + 0.0224	0.9991	0.33	3.4	3			
Naringenin-7-O-glucoside	2.0–40	y = 0.0155x + 0.0063	0.9998	0.11	1.3	4			
Rosmarinic acid	11–123	y = 0.0104x − 0.0268	0.9998	3.7	6.3	1	30/60	80/81	116/98
Apigenin-7-O-glucoside	2.1–83	y = 0.0120x + 0.013	0.9993	0.57	0.95	4			
Salvianolic acid A	2.0–80	y = 0.0160x + 0.0136	0.9977	0.96	5.2	1	6/12		118/102
Eriodictyol	2.1–52	y = 0.0212x + 0.0088	0.9996	0.39	2.3	4			
Scutellarein	0.98–39	y = 0.0212x + 0.0009	0.9986	0.66	2.3	4			
Naringenin	2.0–40	y = 0.0230x + 0.0102	0.9997	0.32	1.8	4			
Luteolin	3.1–78	y = 0.0200x + 0.0064	0.9997	0.38	2.0	1			
Cirsiliol	0.42–2.8	y = 0.0285x − 0.0020	0.9974	0.14	0.30	1			
Kaempferol	1.0–40	y = 0.0130x − 0.0010	0.9999	0.26	0.68	2	7/14	60/65	
Rosmanol	2.2–174	y = 0.0018x − 0.0009	0.9994	0.89	4.1	3			
Apigenin	1.1–46	y = 0.0175x − 0.0060	0.9997	0.35	0.82	1	3/6	94/96	108/103
Cirsimaritin	1.3–60	y = 0.0072x + 0.0004	0.9998	0.45	1.6	1			
Eupatorin	0.96–19	y = 0.0226x − 0.0011	0.9996	0.23	0.65	4	4/8		110/116
Sakuranetin	2–40	y = 0.0201x − 0.0067	0.9999	0.18	1.4	4			
Salvigenin	1.0–60	y = 0.0147x − 0.0247	0.9952	3.7	7.3	5			
Carnosol	2.0–200	y = 0.0023x + 0.0038	0.9985	0.69	3.5	4	30/60		140/135
Carnosic acid	10–200	y = 0.0010x − 0.0062	0.9974	10	19	4	80/160		64/67
Ferulic acid	3.9–90	y = 0.0254x − 0.0046	0.9998	0.54	2.2	2			
*trans*-cinnamic acid	5.9–59	y = 0.0564x − 0.0094	0.9997	0.10	0.71	2			

^a^ y: relative area; x: concentration (µg/mL). ^b^ LOD: limit of detection; LOQ: limit of quantification. ^c^ 1: Sigma–Aldrich (Steinheim, Germany); 2: Fluka (Buchs, Switzerland); 3: Phytolab (Vestenbergsgreuth, Germany); 4: Extrasynthèse (Lyon, France); 5: Targetmol (Wellesley Hills, MA, USA).

**Table 3:** Precision for the phenolic compounds quantified in the solid residue of the EO by-product of *Lamiaceae* aromatic plants studied.

	Repetibility (%RSD, n = 3)				Reproducibility (%RSD, *n* = 9)			
Phenolic Compounds	TM	SL	LL	RO	TM	SL	LL	RO
Phenolic acids								
Danshensu	-	5.0	2.9	5.3	-	6.4	7.4	5.4
Chlorogenic acid	2.2	-	-	2.9	6.5	-	-	3.5
Cryptochlorogenic acid	-	-	-	3.4	-	-	-	6.5
Coumaric acid-O-glucoside	-	-	2.6	-	-	-	8.1	-
p-hydroxybenzoic acid	1.2	-	-	6.1	5.6	-	-	7.6
Ferulic acid-O-glucoside 1	-	-	2.6	-	-	-	7.6	-
Caffeic acid	1.6	2.6	1.0	1.6	3.7	4.7	6.4	3.0
Dihydro-p-coumaric acid glucoside	-	-	6.4	-	-	-	8.1	-
Ferulic acid-O-glucoside 2	-	-	2.2	-	-	-	7.2	-
Rosmarinic acid-3-O-glucoside	-	-	-	4.6	-	-	-	5.4
Rosmarinic acid	2.4	2.1	3.5	2.1	6.4	5.1	7.4	2.3
Salvianolic acid A	-	2.7	2.0	-	-	6.7	6.6	-
Flavones								
6-hydroxyluteolin-7-O-glucoside	2.2	-	-	4.0	5.8	-	-	6.2
Luteolin-7-O-rutinoside	-	2.9	3.8	5.5	-	5.5	5.8	6.2
Luteolin-7-O-glucuronide	-	-	-	4.1	-	-	-	7.6
Luteolin-7-O-glucoside	1.7	3.5	3.3	1.8	6.2	7.5	7.3	4.8
Nepetin 7-O-glucoside	-	-	-	2.0	-	-	-	5.0
Apigenin-7-O-glucoside	1.6	3.8	3.5	-	6.8	6.7	9.3	-
6-hydroxyluteolin	1.1	-	-	-	6.8	-	-	-
Hispidulin-7-O-glucoside	-	-	-	5.3	-	-	-	6.9
6-hydroxyapigenin-7-O-β-glucoside	1.4	-	-	-	7.0	-	-	-
Scutellarein-7-O-glucuronide	-	-	-	2.9	-	-	-	5.2
Scutellarein	3.6	-	-	-	5.9	-	-	-
Luteolin-3′-acetyl-O-glucuronide	-	-	-	2.2	-	-	-	5.2
Luteolin	0.8	3.8	3.5	2.6	4.6	5.1	5.6	3.6
Cirsiliol	-	5.9	-	1.9	-	6.1	-	3.4
Apigenin	0.9	5.4	4.3	1.5	3.7	7.3	8.7	3.1
Eupatorin	-	2.4	-	-	-	4.3	-	-
Cirsimaritin	-	2.1	-	1.5	-	3.9	-	2.6
Ladanein	-	5.1	5.8	1.7	-	5.2	8.7	3.1
Acacetin	-	-	-	2.6	-	-	-	5.5
Genkwanin	-	-	-	3.0	-	-	-	3.2
Salvigenin	-	3.5	-	2.7	-	4.7	-	3.2
4′-methoxytectochrysin	-	-	-	4.5	-	-	-	4.6
Flavanones								
Naringenin-7-O-glucoside	<LQ	-	-	-	<LQ	-	-	-
Hesperidin	-	6.2	-	6.0	-	9.3	-	8.2
Eriodictyol	3.2	-	-	-	5.8	-	-	-
Naringenin	2.6	-	-	-	3.8	-	-	-
Sakuranetin	4.5	-	-	-	5.6	-	-	-
Flavonols								
Kaempferol	1.3	-	-	-	4.0	-	-	-
Phenolic terpene								
Rosmanol	-	-	-	7.3	-	-	-	7.6
(Epi)rosmanol methyl ether	-	-	-	5.5	-	-	-	9.4
Rosmadial	-	-	-	4.7	-	-	-	7.1
Carnosol	-	3.1	-	2.7	-	4.1	-	2.8
Carnosol isomer	-	-	-	3.4	-	-	-	4.0
Carnosic acid derivative	-	-	-	5.3	-	-	-	6.7
Carnosic acid	-	<LQ	-	3.3	-	<LQ	-	5.3
12-methylcarnosic acid	-	-	-	4.0	-	-	-	5.1

LQ: limit of quantification.

**Table 4:** Phenolic compounds quantified in the solid residue of the EO by-product of *Lamiaceae* aromatic plants studied (µg/g of dry weight) ^a^.

Phenolic Compounds	RO	TM	SL	LL	LA	LI_Abrial	LI_Grosso	LI_Super
Phenolic acids								
Danshensu	307 ± 55	-	679 ± 285	1051 ± 451	593 ± 123	879 ± 311	457 ± 146	678 ± 254
Chlorogenic acid	44 ± 34	37 ± 17	-	-	-	-	-	-
Cryptochlorogenic acid	19 ± 7	-	-	-	-	-	-	-
Coumaric acid-O-glucoside	-	-	-	412 ± 163	100 ± 32	161 ± 50	156 ± 33	195 ± 28
p-hydroxybenzoic acid	16 ± 10	35 ± 18	-	-	-	-	-	-
Ferulic acid-O-glucoside 1	-	-	-	66 ± 30	131 ± 44	167 ± 58	132 ± 29	147 ± 28
Caffeic acid	88 ± 14	108 ± 32	123 ± 31	54 ± 19	79 ± 14	63 ± 18	114 ± 46	93 ± 54
Dihydro-p-coumaric acid glucoside	-	-	-	244 ± 97	69 ± 12	116 ± 37	107 ± 20	104 ± 22
Ferulic acid-O-glucoside 2	-	-	-	57 ± 29	158 ± 54	193 ± 75	164 ± 32	154 ± 37
Rosmarinic acid-3-O-glucoside	421 ± 284	-	-	-	-	-	-	-
Rosmarinic acid	2283 ± 720	1997 ± 1158	1486 ± 670	599 ± 272	579 ± 173	343 ± 109	235 ± 70	290 ± 67
Salvianolic acid A	-	-	457 ± 273	596 ± 304	252 ± 98	285 ± 135	282 ± 98	385 ± 113
Flavones								
6-hydroxyluteolin-7-O-glucoside	40 ± 38	1600 ± 751	-	-	-	-	-	-
Luteolin-7-O-rutinoside	61 ± 63	-	298 ± 161	158 ± 75	91 ± 20	150 ± 61	122 ± 23	146 ± 32
Luteolin-7-O-glucuronide	99 ± 91	-	-	-	-	-	-	-
Luteolin-7-O-glucoside	79 ± 67	2235 ± 903	195 ± 84	385 ± 118	14 ± 13	52 ± 19	45 ± 13	62 ± 20
Nepetin 7-O-glucoside	352 ± 190	-	-	-	-	-	-	-
Apigenin-7-O-glucoside	-	793 ± 285	426 ± 179	273 ± 70	153 ± 21	255 ± 98	162 ± 32	133 ± 72
6-hydroxyluteolin	-	440 ± 242	-	-	-	-	-	-
Hispidulin-7-O-glucoside	302 ± 62	-	-	-	-	-	-	-
6-hydroxyapigenin-7-O-β-glucoside	-	215 ± 75	-	-	-	-	-	-
Scutellarein-7-O-glucuronide	584 ± 151	-	-	-	-	-	-	-
Scutellarein	-	201 ± 155	-	-	-	-	-	-
Luteolin-3′-acetyl-O-glucuronide	364 ± 122	-	-	-	-	-	-	-
Luteolin	131 ± 43	636 ± 323	157 ± 52	42 ± 24	30 ± 10	49 ± 22	64 ± 17	60 ± 22
Cirsiliol	56 ± 37	-	20 ± 14	-	-	-	-	-
Apigenin	195 ± 82	665 ± 233	145 ± 61	29 ± 10	95 ± 35	100 ± 44	81 ± 21	64 ± 18
Eupatorin	-	-	95 ± 48	-	-	-	-	-
Cirsimaritin	1032 ± 509	-	382 ± 328	-	-	-	-	-
Ladanein	234 ± 141	-	74 ± 71	46 ± 14	93 ± 33	72 ± 20	93 ± 25	70 ± 18
Acacetin	127 ± 100	-	-	-	-	-	-	-
Genkwanin	606 ± 339	-	-	-	-	-	-	-
Salvigenin	398 ± 239	-	531 ± 343	-	-	-	-	-
4′-methoxytectochrysin	692 ± 308	-	-	-	-	-	-	-
Flavanones								
Naringenin-7-O-glucoside	-	87 ± 85	-	-	-	-	-	-
Hesperidin	380 ± 148	-	41 ± 86	-	-	-	-	-
Eriodictyol	-	311 ± 132	-	-	-	-	-	-
Naringenin	-	310 ± 94	-	-	-	-	-	-
Sakuranetin	-	279 ± 103	-	-	-	-	-	-
Flavonols								
Kaempferol	-	268 ± 147	-	-	-	-	-	-
Phenolic terpene								
Rosmanol	218 ± 190	-	-	-	-	-	-	-
(Epi)rosmanol methyl ether	657 ± 434	-	-	-	-	-	-	-
Rosmadial	189 ± 205	-	-	-	-	-	-	-
Carnosol	6862 ± 1511	-	1055 ± 2203	-	-	-	-	-
Carnosol isomer	1809 ± 1605	-	-	-	-	-	-	-
Carnosic acid derivative	6937 ± 2416	-	559 ± 1703	-	-	-	-	-
Carnosic acid	26,703 ± 9259	-	867 ± 2687	-	-	-	-	-
12-Methylcarnosic acid	4174 ± 1789	-	1769 ± 4541	-	-	-	-	-
Total phenolic compounds	56,459 ± 1375	10,002 ± 3193	9301 ± 11,089	4015 ± 1343	2436 ± 463	2884 ± 859	2213 ± 341	2582 ± 431

^a^ means ± standard deviation.

## Data Availability

Data are contained within the article and Supplementary Materials.

## Supplementary Materials

The following supporting information can be downloaded at https://www.mdpi.com/article/10.3390/molecules29225285/s1. Table S1: Factor loadings after varimax rotation of all the samples of solid residues obtained from the distillation of the MAPs; Table S2: Factor loadings after varimax rotation of the Lavandula spp. samples of solid residues obtained from the distillation.
