# Supplementary material for: Characterization of Bioactive Phenolic Compounds Extracted from Hydro-Distillation By-Products of Spanish Lamiaceae Plants
Source: Molecules. 2024 Nov 8;29(22):5285. doi: 10.3390/molecules29225285 (PMC11596117; doi:10.3390/molecules29225285)
Supplement: Supplementary file 1 [file molecules-29-05285-s001.zip › molecules-3261469-supplementary.pdf]

Table S1. Factor loadings after varimax rotation of all the samples of solid residues obtained from the distillation of the MAPs.

**Phenolic compounds**	**F1**	**F2**	**F3**	**F4**
Danshensu	0.282	-0.773		
Chlorogenic acid		0.867		
Cryptochlorogenic acid	0.845	0.414		
Coumaric acid-O-glucoside		-0.552	-0.550	
p-hydroxybenzoic acid	-0.325	0.731		
Ferulic acid-O-glucoside 1		-0.562	-0.588	
Caffeic acid			0.564	
Dihydro-p-coumaric acid glucoside		-0.560	-0.561	
Ferulic acid-O-glucoside 2		-0.532	-0.561	
Rosmarinic acid-3-O-glucoside	0.735	0.368		
Rosmarinic acid		0.608	0.418	
Salvianolic acid A		-0.742	0.262	
6-hydroxyluteolin-7-O-glucoside	-0.550	0.710		
Luteolin-7-O-rutinoside		-0.616	0.575	
Luteolin-7-O-glucuronide	0.677	0.330		-0.346
Luteolin-7-O-glucoside	-0.583	0.689		
Nepetin 7-O-glucoside	0.815	0.402		-0.307
Apigenin-7-O-glucoside	-0.667	0.502		
6-hydroxyluteolin	-0.556	0.709		
Hispidulin-7-O-glucoside	0.882	0.421		
Scutellarein-7-O-glucuronide	0.861	0.406		
Scutellarein	-0.488	0.620		
Luteolin-3′-acetyl-O-glucuronide	0.826	0.382		0.276
Luteolin	-0.497	0.683		
Cirsiliol	0.752			-0.365
Apigenin	-0.492	0.746		
Eupatorin		-0.252	0.733	
Cirsimaritin	0.731		0.257	0.269
Ladanein	0.794			
Acacetin	0.707	0.337		
Genkwanin	0.793	0.381		
Salvigenin	0.439		0.739	
4′-methoxytectochrysin	0.823	0.405		
Naringenin-7-O-glucoside	-0.420	0.539		
Hesperidin	0.850	0.415		
Eriodictyol	-0.583	0.735		
Naringenin	-0.595	0.728		
Sakuranetin	-0.578	0.707		
Kaempferol	-0.511	0.616		
Rosmanol	0.607	0.262		0.646
(Epi)rosmanol methyl ether	0.734	0.331		
Rosmadial	0.648	0.326		-0.587

Carnosol	0.815		0.259	
Carnosol isomer	0.714	0.355		-0.510
Carnosic acid derivative	0.817	0.297		
Carnosic acid	0.861	0.367		
12-Methylcarnosic acid	0.479		0.485	
Variance (%)	36.3	26.2	8.5	4.1
Cumulative variance (%)	36.3	62.5	71.0	75.1

Loadings lower than absolute values of 0.250 are not shown

Table S2. Factor loadings after varimax rotation of the *Lavandula* spp. samples of solid residues obtained from the distillation.

**Phenolic compounds**	**F1**	**F2**	**F3**
Danshensu	0.819	0.310	-0.275
Coumaric acid-O-glucoside	0.905		
Ferulic acid-O-glucoside 1	-0.587	0.632	-0.343
Caffeic acid	-0.418	0.647	
Dihydro-p-coumaric acid glucoside	0.885		
Ferulic acid-O-glucoside 2	-0.691	0.558	-0.351
Rosmarinic acid	0.790		
Salvianolic acid A	0.737	0.265	
Luteolin-7-O-rutinoside	0.567	0.721	
Luteolin-7-O-glucoside	0.936		
Apigenin-7-O-glucoside	0.721	0.292	-0.320
Luteolin		0.687	0.626
Apigenin	-0.665	0.530	
Ladanein	-0.676		-0.283
Variance (%)	50.5	20.3	7.4
Cumulative variance (%)	50.5	70.9	78.3

Loadings lower than absolute values of 0.250 are not shown
